# Our Bureau of Information

**Published:** 1912-03-16

**Authors:** 


### OUR BUREAU OF INFORMATION.

### On Hastening Slowly.

It may not be amiss to explain to our correspondents why their questions and letters are not always dealt with immediately. So far as the information at our command allows, queries are answered in the first possible issue—that is, within ten days, though occasionally the pressure on our space may necessitate postponement for a week. But sometimes questions are sent which must be referred to specialists, or which take time to investigate. They are published in our page, and if an answer comes from one of our readers we are very grateful. If not, we try to find out for ourselves—a business which may take weeks. We wish it to be understood that a query is not ignored because it is published without an answer attached. On the contrary, we are seeking for the best sources of information.

But in the matter of mutual help there is often still greater difficulty. We publish an appeal, and an answer comes to us. It would seem to be the easiest thing in the world to bring our two correspondents into communication. But this involves us in a certain responsibility, and we want to fulfil it rightly. We must make sure of the *bona fides* of people. If a young girl asks for a home and one is offered to her, it is our duty to make sure that the home is one to which a mother could safely entrust her daughter. There is another side to the story. We do not want to send to even the kindest and most charitable a guest who would be socially unacceptable. People are happiest with those of their own class, and it is no kindness to send them as guests to persons in a different station.

Even offers of service must be investigated. We are grateful to all who wish to help us, but we must have some guarantee of suitability. Moreover we have to ask those who want to do "something" what is the something that they wish to do. Do they wish to visit in hospitals, and if so where? If we know that, we can suggest to what institutions they ought to apply, with the proviso that it rests with the officials there to accept or refuse their services. When the offer is to do work outside, a more individual responsibility is ours. It is not easy to say of a person whom one has never seen that he or she should undertake certain work. We can but publish the offer, and if there is any response try to find out what help would be welcome in the particular case. The ultimate arrangement must be between the parties themselves, but it is our business to avoid, in so far as we can, making introductions which would in the end prove futile.

Of course, some of our correspondents resent this care. Conscious of their own integrity, they regard inquiries as an impertinence. It is unfortunate, but we cannot help that. Let our readers realise that the precautions we take, however unnecessary they may be in their case, may be essential in others, and they will not judge our carefulness harshly.

### Needs and Helpers.

Cheap Home for an Old Lady.

"S. A. L." writes: I wonder if you could help me in this matter, viz.: Do you know of anyone in London, nurse or otherwise, who would offer a kind home to an old lady (70) in very reduced circumstances? Twelve shillings weekly offered. Institution or Home not wished for. Perhaps I ought to state that she suffers from bronchitis and is not able to walk much.

### Answers to Correspondents.

"A. L. B." writes: How does one get a person into a Home for Inebriates?

[It is very difficult to "get" anyone into such a Home, or retreat, as they are called. In the first place the application for admission must come from the victim of the vice himself, and when making it he must promise to conform to the rules of the institution which he enters, and specify the time he will remain. This application must be accompanied by a declaration from two persons stating not only that the person concerned is a habitual drunkard, but that he understood the meaning and result of the application, and the signature must be attested by two justices of the peace, who must also satisfy themselves on these points. It will thus be seen that admission depends on the unfortunate inebriate being willing to go into the retreat. When, however, these formalities are successfully carried through, he cannot leave the retreat until the time he has signed for has elapsed, and many inebriates, in their better moments, are willing to accept the prolonged discipline necessary for their reformation.]

"Elise" writes: I am a clerk (shorthand and typing). My health has not been good, and I am recommended to go to South Africa. What part should I go to and what are the chances of my obtaining work out there? I cannot afford to remain idle.

[Unless you have friends, it would be rash to go out without taking steps to secure a post immediately on your arrival. You would have more chance of obtaining occupation as a domestic servant than as a clerk, and as kaffirs do all the rough work, the position need not be distasteful to an educated woman. Many ladies would be glad to get such a one to look after their children. The Cape Colony is the best for delicate lungs, but both the Transvaal and the Orange River Colony are dry and healthy, though cold in the winter. The inland parts of Natal are healthy, but in Durban and around the coast generally it is hot and trying for English people. You can get detailed information from the Emigration Information Office, 31 Broadway, Westminster, S.W.]

"Mater" writes: My daughter is coming to London to a situation. We have no friends there, and I am anxious about letting her go so far alone. But I cannot afford to come with her. Can you help me?

[Write to the Travellers' Aid Society, 3 Baker Street, London, W. A representative of the Society would meet your daughter on her arrival and see her safely to her destination. Unless you know the people to whom your daughter is going, it might be well to ask the Society to make some inquires about them.]

### Rules for Correspondents.

Everyone who uses or wishes to help our Bureau of Information must be kind enough to observe the following rules:—

(1) Postal replies cannot be given. Exception will only be made in very special cases at the Editor's discretion.

(2) Queries or answers relating to different cases must be written, or preferably typed, on separate sheets of paper, and the writing must never be crossed.

(3) Questions and replies received not later than Wednesday will, as far as possible, be dealt with in our issue of the following Saturday week.

(4) Letters must have attached the name and address of the sender, with a pseudonym for publication if preferred.

(5) Applications referring to non-dependents where the need relates to business or employment must be accompanied by a postal order for 5s., when the requirement shall have publicity in these columns.

(6) All applicants for insertion in our list of Approved Homes must send a registration fee of five shillings. The omission of this leads to delay and gives avoidable trouble.

(7) *All communications* for this department *must be accompanied by the coupon* to be found at the bottom of the third page of the cover on the inside, and should be addressed to the Editor of the Bureau of Information, c/o The Hospital, 29 Southampton Street, Strand, London, W.C.

